# Disaster Preparedness and the Chronic Disease Needs of Vulnerable Older Adults

**Published:** 2007-12-15

**Authors:** Nancy Aldrich, William F Benson

**Affiliations:** Aging Information Specialist, AgingOpportunities.com; Principal, Health Benefits ABCs, Silver Spring, Maryland

## Abstract

About 80% of older adults have at least one chronic condition that makes them more vulnerable than healthy people during a disaster. These chronic conditions — combined with the physiological, sensory, and cognitive changes experienced as part of aging — result in frail older adults having special needs during emergencies. Planning and coordination among public health and emergency preparedness professionals and professionals who provide services for the aging are essential to meet these special needs. Several tools and strategies already exist to help prepare these professionals to protect and assist older adults during a disaster. These include having professionals from diverse fields work and train in coalitions, ensuring that advocates for older adults participate in community-wide emergency preparedness, and using community mapping data to identify areas where many older adults live.

## Introduction

An estimated 14 million people aged 65 or older living outside an institution reported in Census 2000 that they had some level of disability, mostly linked to chronic conditions such as heart disease or arthritis (1). Frail older adults — defined as those with serious, chronic health problems — are more likely than the healthier or younger population to need extra assistance to evacuate, survive, and recover from a disaster (2). In fact, at least 13 million older adults (aged 50 years or older) in the United States have said they would need help to evacuate during a disaster, and about half of these would require help from someone outside their household (3).

Disasters disproportionately affect frail older adults. Before hurricanes Katrina and Rita in 2005, adults aged 60 or older made up only 15% of the population of New Orleans, Louisiana (4,5). However, 71% of those who died because of the hurricane were over age 65 (6). During the 1995 heat wave in the Midwest, the median age of the 465 people in Chicago whose deaths were heat-related was 75 (7).

The sheer numbers of the aging population give an even greater urgency to addressing the needs of older adults following a disaster. The U.S. population aged 65 or older is expected to almost double in size within the next 25 years (8). By 2030, some 72 million people — almost one of every five Americans — will be aged 65 or older (1). People aged 85 or older are the fastest growing segment of the U.S. population (1).

## Chronic Disease and Disability

Arthritis, hypertension, heart disease, diabetes, and respiratory disorders are some of the leading causes of activity limitations among adults aged 65 or older (1). These conditions can impair an older adult's ability to prepare, respond, or recover from a disaster. Treating chronic disease following a natural disaster must, therefore, become a public health and medical priority (9). Emergency managers who work with public health and providers of services for the aging (aging services) need to place a priority on special planning for frail older adults who encounter severe weather-related events, earthquakes, large-scale attacks on civilian populations, technological catastrophes, influenza pandemics, or other disasters.

Consider the following statistics:

- About 80% of adults aged 65 or older have at least one chronic health condition (1).
- About 50% of older adults have at least two chronic conditions (1).
- Nearly 50% of adults aged 65 or older have hypertension, 36% have arthritis, 20% have coronary heart disease, 20% have cancer, 15% have diabetes, and 9% have had a stroke (10).

Chronic conditions often lead to disabilities and the inability to perform basic activities of daily living (ADLs) such as bathing, dressing, eating, and moving around the house. In 2002, 52% of older adults reported that they had some type of disability, including 37% who reported a severe disability and 16% who reported that they needed some type of assistance as a result of their disability (11). In 2004, another study found that about 27% of community-dwelling Medicare beneficiaries older than age 65 reported that they had difficulty in performing one or more ADLs, and an additional 13.7% reported difficulties with other activities such as preparing meals or shopping (11).

After a disaster, conditions such as stress, the lack of food or water, extremes of heat or cold, and exposure to infection can contribute to rapid worsening of a chronic illness that was under control before the event (12). Interruptions in medication regimens and needed medical technologies also can exacerbate underlying conditions and increase the risk of morbidity or mortality (12,13). Older adults with chronic conditions also may face health risks from either inadequate nutrition or from too much sodium, fat, and calories contained in the Meal, Ready-to-Eat (MRE) packages often offered to evacuees.

Following Hurricane Katrina, a survey of 680 evacuees living in Houston shelters in September 2005 showed that 41% reported having chronic health conditions such as heart disease, hypertension, diabetes, and asthma; 43% indicated that they were supposed to be taking a prescription medication; and 29% of those who were supposed to take prescription drugs said they had problems getting prescriptions filled (14). Most of those surveyed did not give their age, but many of the people who were in shelters were older adults (14).

## Special Characteristics of Older Adults

Certain characteristics of older adults may prevent them from adequately preparing for disasters and may hinder their adaptability during disasters. In addition to chronic health conditions, older adults may have impaired physical mobility or cognitive ability, diminished sensory awareness, and social and economic limitations (2). For example, declining vision or hearing can make it difficult for an older adult to communicate. Older adults with cognitive problems may become agitated during a crisis or feel overwhelmed by the crowding, noise, and lack of privacy in a shelter. They may need assistance to ensure that they have their medications, adequate nutrition and water, and assistive devices. Older adults also may be more vulnerable to emotional trauma during a disaster (15). Because older adults are often reluctant to seek or accept mental health services, they may not obtain the counseling they need, even if it is available (13,16).

## Lessons Learned

The public health role following disasters traditionally has focused on preserving lives; ensuring safe food, water, and sewage disposal; and controlling infectious disease, environmental risks, and pests. Only rarely was there a need to take any special action for older adults with chronic conditions, because people generally were quickly able to return to normal after a short-duration disaster. In these circumstances, chronic disease did not seem to be a public health or medical priority (9). The September 11, 2001, terrorist attack in New York City created a greater awareness of the needs of the chronically ill population, but it was not until the catastrophic hurricanes that struck the Gulf Coast in 2005 that public health and other professionals fully grasped the urgency of addressing the chronic health needs of vulnerable populations during disasters. The destruction of the medical infrastructure, the displacement of residents from their homes, and the inability to access pharmacies or medical care all contributed to the emergence of chronic diseases as a critical concern (9).

On September 11th, about 6,300 seniors lived around the World Trade Center's Twin Towers in New York City, and nearly 19,000 older adults lived within a three-block radius (17). Many frail older adults and persons with disabilities were confined for days to their high-rise apartments near the World Trade Center without electricity, fresh supplies, a way to refill their medications, or any way to communicate with the outside world (18). Home care workers could not get in to visit their clients (17), and community service providers could not get to their offices or access computers with client information. In addition, many frail adults were unknown to community workers because they had never applied for services (18).

Along the Gulf Coast in 2005, hurricanes Katrina and Rita and the accompanying flooding resulted in 1,330 deaths, many of which were among older adults (3). In addition, an estimated 200,000 people with chronic medical conditions, who were evacuated or isolated after Hurricane Katrina, lacked access to their medications and usual sources of care (6). As the recovery effort continued, even those evacuees who had the recommended three-day supply of prescriptions ran out.

Since Hurricane Katrina, public health personnel, emergency responders, and aging services professionals have begun working together to plan for protecting frail older adults who may need assistance following a disaster. The goal is to create an emergency response system that can rescue and shelter vulnerable populations and then ensure that they continue to receive routine health care, such as prescription medications, as recommended by the Chronic Diseases and Vulnerable Populations in Natural Disasters Working Group, part of the Coordinating Center for Health Promotion, Centers for Disease Control and Prevention (CDC) (12).

## Recommendations

In response to September 11th and to the Gulf Coast hurricanes of 2005, experts have made recommendations to communities on preparing for disaster:

- Develop strong relationships and partnerships between public health agencies, services for the aging, emergency responders, and other entities before disaster strikes to improve coordination, communication, and response in emergency situations (3).
- Have backup communications systems, and maintain a copy of essential information in two locations (18).
- Use mapping systems to identify areas with high concentrations of older adults (18).
- Create a citywide emergency plan for older adults and people with disabilities (18) that includes a separate shelter area for them (19), an evacuation system that includes transporting their medications and supplies with them, a network of emergency pharmaceutical services (17), and a system for evacuating pets (6).
- Provide appropriate public information on emergency preparedness in appropriate formats to older adults and people with disabilities (3).
- Establish a secure system of photo identification and permits for professional health care and senior service workers that will enable them to reach their homebound clients in an emergency (17,18).
- Develop an emergency support system for in-home services, including emergency respite care and communications systems for in-home caregivers (17).
- Create a list of volunteers willing to help in an emergency (17).
- Arrange with local restaurants to provide food to older adults during an emergency (17).
- Improve identification and tracking methods for older adults and their health information (3).

Resources to aid communities in addressing these recommendations and Internet addresses for these resources are listed in the Table.

## Working With the Aging Services Network

Public health professionals can create the most effective disaster preparedness plans for vulnerable adults by working with the network of aging services professionals (known as the "aging services network"), which includes state and local departments on aging, local service providers, and Indian tribal organizations that provide services to older adults. The network, operating under the auspices of the federal Older Americans Act, already plays a vital role in delivering meals and providing transportation, information, and other services to older adults. During a disaster, this network reaches out to its clients and identifies those who need assistance obtaining food, water, shelter, or medications (20).

## Tools for Preparedness Planning

### Surveillance and assessment

Community assessments following disasters can identify health-related needs and support public health interventions (21). CDC can help state and local public health agencies use existing health surveillance systems to estimate the need for emergency responders who can address chronic health conditions and disabilities following a disaster.

Following the events of September 11th, Connecticut, New Jersey, and New York added a mental health module to their ongoing Behavioral Risk Factor Surveillance System (BRFSS) surveys to help public health professionals understand the importance of addressing the physical and emotional needs of older adults living in the area (22). BRFSS data and data from other information systems provide information on the prevalence of diabetes, heart disease, stroke, hypertension, and asthma before a disaster, giving planners better knowledge about the needs of their population with chronic disease (9).

### Geographic mapping systems

A geographic information system can map the residences of older adults and persons with special needs who will require assistance during an emergency evacuation. After Hurricane Charley crossed Florida in 2004, CDC provided population maps for the three most damaged counties to enable workers to identify and interview someone from almost 600 households with an older adult (23). In one county, workers found that in one-third of the households with a chronically ill older adult, at least one of the older person's conditions had worsened because of the hurricane; 28% of the households reported that an older adult was unable to receive routine care for a chronic disease. In another county, 9% of households with older adults did not have access to prescription drugs. Local health care providers used this information to accelerate restoration of medical services and access to medications in the affected areas (23).

### Handbooks

The U.S. Administration on Aging's *Emergency Assistance Guide 2006* helps professionals plan for emergencies (Table). In addition, the American Red Cross has materials that focus on special populations, including *Disaster Preparedness for Seniors by Seniors* and *Disaster Preparedness for People with Disabilities* (Table). The Florida International University and University of South Florida, with funding from the U.S. Administration on Aging, have developed a planning tool for aging services professionals. The tool, titled *Designing a Model All-Hazards Plan for Older Adults: The Role of the Aging Services Network in Assuring Community All-Hazards Readiness for Elders and in Providing Assistance to Elders when Disasters Occur* (Table), contains detailed recommendations on addressing the needs of vulnerable older adults in all areas of the country during disasters. CDC's Coordinating Office for Terrorism Preparedness and Emergency Response has drafted *Public Health Workbook to Define, Locate and Reach Special, Vulnerable, and At-Risk Populations in an Emergency* (Table).

## Conclusion

Planning for assisting populations with chronic diseases, especially vulnerable older adults, during a disaster is essential to meeting their special needs. Public health professionals should link with professionals in aging services, emergency planning, and other groups to create a comprehensive system for addressing the needs of older adults during a disaster. Planning, coalition building, and using mapping systems are among the numerous tools and strategies available to creating an emergency response system that can rescue and shelter vulnerable populations in disaster situations.

## Figures and Tables

**Table:** Resources Available to Help Communities Prepare for Disasters

Resource	Responsible Agency or Organization	Web link
Behavioral Risk Factor Surveillance System	Centers for Disease Control and Prevention (CDC)	http://www.cdc.gov/brfss/
*CDC's Disaster Planning Goal: Protect Vulnerable Older Adults*	CDC (through a contract with Health Benefits ABCs)	http://www.cdc.gov/aging/pdf/disaster_planning_goal.pdf
*Designing a Model All-Hazards Plan for Older Adults: The Role of the Aging Services Network in Assuring Community All-Hazards Readiness for Elders and in Providing Assistance to Elders when Disasters Occur *	Florida International University and University of South Florida (Administration on Aging contract)	http://www.allianceforaging.org/pdfs/DisasterPlan.pdf
Disaster assistance Web site	Administration on Aging	http://www.aoa.gov/ELDFAM/Disaster_Assistance/Disaster_Assistance.asp
*Disaster Planning Tips for Older Adults and Their Families*	CDC (contract with Health Benefits ABCs)	http://www.cdc.gov/aging/pdf/disaster_planning_tips.pdf
*Disaster Preparedness for People with Disabilities*	American Red Cross	http://www.prepare.org/disabilities/disabilities.htm
*Disaster Preparedness for Seniors by Seniors*	American Red Cross	http://www.redcross.org/services/disaster/0,1082,0_9_,00.html
*Eldercare Locator *(provides links to aging network resources)	Administration on Aging	http://www.eldercare.gov
*Emergency Assistance Guide 2006*	Administration on Aging	http://www.aoa.gov/PRESS/preparedness/preparedness.asp#guide
*Emergency Preparedness Tips for Older Adults*	Foundation for Health in Aging, American Geriatrics Society	http://www.healthinaging.org/public_education/disaster_tips.pdf
*Just in Case: Emergency Readiness for Older Adults and Caregivers*	Administration on Aging	http://www.aoa.gov/PROF/aoaprog/caregiver/overview/Just_in_Case030706_links.pdf
*Pandemic Flu Operational Plan*	Administration on Aging	http://www.aoa.gov/press/preparedness/pdf/AoA Flu Pandemic Draft Plan 7-20-06a.doc
*Preparing for Disaster for People with Disabilities and Other Special Needs*	Federal Emergency Management Agency and the American Red Cross	http://www.fema.gov/pdf/library/pfd_all.pdf
*Public Health Workbook to Define, Locate and Reach Special, Vulnerable, and At-Risk Populations in an Emergency *(draft)	CDC	http://www.bt.cdc.gov/workbook/
Ready America Web site	U.S. Department of Homeland Security	http://www.ready.gov/america/getakit/seniors.html
*Resources for Planning How to Protect Your Pets in an Emergency*	CDC	http://www.bt.cdc.gov/disasters/petprotect.asp
